# The Role of Selenium in the Antioxidant System of Cattle, Pigs, and Small Ruminants: Implications for Animal Health and Productivity

**DOI:** 10.3390/ani16071019

**Published:** 2026-03-26

**Authors:** Katarzyna Żarczyńska, Katarzyna Różańska, Oliwia Świerczek, Dawid Tobolski

**Affiliations:** 1 Department and Clinic of Internal Diseases, Faculty of Veterinary Medicine, University of Warmia and Mazury in Olsztyn, 10-719 Olsztyn, Poland; katarzyna.zarczynska@uwm.edu.pl (K.Ż.); 157891@student.uwm.edu.pl (K.R.); 2 Department of Large Animal Diseases and Clinic, Institute of Veterinary Medicine, Warsaw University of Life Sciences, 02-787 Warsaw, Poland; s218219@sggw.edu.pl

**Keywords:** selenium, selenoproteins, oxidative stress, antioxidant defense, dairy cattle, pigs, small ruminants, organic selenium, trace mineral supplementation

## Abstract

Oxidative stress contributes to reproductive disorders, immune dysfunction, and reduced productivity in livestock during periods of high metabolic demand and environmental challenge. Selenium supports antioxidant defense systems because it is incorporated as selenocysteine into selenoproteins, including glutathione peroxidases and thioredoxin reductases that detoxify peroxides and sustain redox balance. The review summarizes selenium occurrence and chemical forms in feeds, as well as its absorption, transportation, and storage. The review also outlines the major features of selenoprotein biosynthesis and its prioritized allocation, with an emphasis on cattle, pigs, sheep, and goats. Evidence from multiple sources indicates that selenium status and supplementation interacts with antioxidant capacity, immune competence, thyroid hormone metabolism, reproductive performance, and the transfer of selenium to milk and offspring. In ruminants, rumen microbial transformations can reduce the bioavailability of inorganic selenium salts, and organic sources, such as selenium-enriched yeast, hydroxy-selenomethionine, and selenitetriglycerides, often increase blood and milk selenium more effectively. In pigs, organic selenium is commonly associated with enhanced antioxidant and immune indices in sows and piglets during late gestation, lactation, and weaning, whereas effects on growth performance are inconsistent. The review emphasizes the narrow margin between adequacy and excess and outlines practical considerations for supplementation and monitoring, alongside research needs for emerging selenium forms and functional biomarkers.

## Simple Summary

Livestock often experience oxidative stress when reactive molecules exceed antioxidant defenses and damage cells. Oxidative stress can impair fertility, immunity, growth, and product quality, particularly around parturition, weaning, rapid growth, heat stress, and infection. Selenium is a trace mineral needed to build selenium-containing proteins that control peroxides and support immune and hormone function. Dietary selenium supply varies with soil and feed ingredients, so supplementation is common, but the safe range between deficiency and excess is narrow. The review explains how the form of selenium influences the absorption, storage, and biological effects in cattle, pigs, sheep, and goats. Available evidence indicates that adequate selenium supports antioxidant capacity and resilience to disease, whereas deficiency increases risk of conditions such as white muscle disease in young ruminants. In cattle, sheep, and goats, organic sources such as selenium-enriched yeast often improve selenium status and milk selenium concentration more than mineral salts because the rumen can convert mineral forms into poorly absorbed compounds. The review concludes that species- and stage-specific supplementation, supported by monitoring selenium status, can improve animal health, productivity, and product quality.

## 1. Introduction

Reactive oxygen species (ROS) and reactive nitrogen species (RNS) are generated continuously in aerobic organisms as both unavoidable byproducts of metabolism and deliberately produced mediators of cellular signaling. In redox biology, ROS and RNS are increasingly discussed as an integrated chemical and functional continuum because many biologically relevant oxidants arise from reactions that couple oxygen- and nitrogen-centered intermediates. The umbrella term reactive oxygen and nitrogen species (RONS) captures this overlap [1,2]. Under homeostatic conditions, RONS act as spatially and temporally restricted signals that modulate transcription, proliferation, differentiation, glucose handling, and programmed cell death, largely through reversible oxidation of redox-sensitive protein residues and regulation of kinase–phosphatase networks [2]. Nitric oxide (NO), classically categorized as an RNS, exemplifies this duality by acting as a physiological signaling mediator while also serving as a precursor of secondary nitrating and oxidizing species in inflamed or metabolically stressed tissues [1].

The biological impact of RONS reflects on the balance between their production, compartmentalization, and removal. Endogenous ROS arise from mitochondrial electron transport, oxidase activity at cellular membranes, and redox reactions in organelles that sustain lipid and protein biosynthesis. Reactive nitrogen intermediates are generated predominantly via NO synthase pathways, and their downstream chemistry is shaped by local ROS availability, particularly the rapid coupling of NO with superoxide to form potent oxidants. These intersecting reactions mean that changes in mitochondrial function, oxygen delivery, nutrient flux, or inflammatory activation can rapidly alter oxidant profiles within the tissue microenvironment [1,2,3]. A prominent example of regulated ROS generation is innate immune defense, where phagocytes employ an oxidative burst to support killing of invasive microorganisms. This antimicrobial strategy is effective but intrinsically hazardous to host tissues and to immune cells themselves, making adequate antioxidant capacity a prerequisite for efficient pathogen clearance without excessive collateral injury [4].

Oxidative stress is commonly defined as a sustained imbalance in which oxidant formation exceeds antioxidant and repair capacity, resulting in a net shift toward oxidation [5]. Importantly, oxidative stress does not reflect oxidant load alone; it also reflects whether cellular buffering systems can regenerate reducing equivalents, repair oxidized macromolecules, and restore redox-sensitive signaling to basal levels. When oxidative pressure persists, damage accumulates in lipids, proteins, and nucleic acids, thereby compromising membrane integrity, enzyme activity, receptor function, and genomic stability [6]. Such molecular injury can translate into clinically relevant phenotypes, including impaired immune responsiveness, reproductive dysfunction, reduced growth, and increased susceptibility to infectious and metabolic disease, especially when tissue repair pathways are inefficient or chronically overloaded [2,7,8].

These mechanisms are particularly relevant to livestock because modern production systems combine high metabolic demand with repeated environmental and management stressors. Thermal extremes, pathogen pressure, dietary transitions, and exposure to environmental toxicants all shape redox balance, often acting concurrently rather than in isolation. In intensive systems, the co-occurrence of heat stress, high stocking density, and nutritional imprecision can amplify oxidative injury and contribute to impaired welfare and reduced production efficiency [9,10,11]. Physiological states such as the periparturient period in dairy cattle, rapid postnatal growth, and high-output lactation are characterized by high oxygen flux and dynamic endocrine and metabolic remodeling, which together increase reliance on robust antioxidant buffering to preserve tissue function [8,10]. In this context, oxidative stress is not merely a biochemical descriptor; it is a cross-cutting contributor to performance-limiting disease and subclinical inefficiency that affects reproduction, immunity, and product quality [7,8,12].

To control RONS while preserving their signaling roles, organisms rely on an integrated antioxidant system comprising enzymatic and non-enzymatic components. This network functions across cellular compartments and can be conceptualized as a layered defense: catalytic enzymes detoxify primary oxidants, chain-breaking molecules buffer radicals and lipid peroxyl intermediates, and downstream pathways remove or repair oxidation products [13]. The enzymatic arm includes superoxide dismutase (SOD), catalase (CAT), glutathione peroxidases (GPx), and thioredoxin reductases (TrxR), which together limit the accumulation of superoxide and peroxides and maintain thiol redox homeostasis that underpins protein function and redox signaling [4,14,15]. These defenses are especially prominent in organs with high oxygen demand and high metabolic activity, reflecting the need to stabilize redox balance in tissues that continuously manage detoxification, nutrient processing, and energy metabolism [16]. Enzymatic capacity is not autonomous; it depends on adequate micronutrient supply because copper, zinc, iron, manganese, and selenium serve as essential cofactors or structural elements of antioxidant enzyme active sites [17]. Consequently, trace element insufficiency can convert otherwise tolerable metabolic or inflammatory RONS flux into clinically meaningful oxidative stress. Peroxiredoxins and the thioredoxin system, including thioredoxin and thioredoxin reductases, further extend peroxide detoxification and thiol redox cycling within the same antioxidant network [2,14,15].

Non-enzymatic antioxidants complement enzymatic defenses by providing rapid buffering in aqueous and lipid compartments and by supporting regeneration cycles that sustain reducing power. This group includes vitamins A, C, and E, coenzyme Q10, flavonoids, and reduced glutathione (GSH), with GSH occupying a central position as both a direct redox buffer and a substrate for peroxide detoxification by GPx [18,19]. Antioxidant availability is determined by dietary intake, endogenous biosynthesis, and regeneration pathways, and imbalances between enzymatic activity and non-enzymatic buffering can increase vulnerability to oxidative injury even when total oxidant production is unchanged [16]. Beyond preventing structural damage, the antioxidant system also constrains redox-driven transcriptional pathways, thereby influencing immune function and apoptosis through redox-sensitive gene regulation [16].

Within this network, selenium (Se) is distinctive because its principal biological actions are mediated through selenoproteins, in which selenium is incorporated as selenocysteine (Sec) into catalytic motifs of redox enzymes [20,21]. Selenium-dependent enzymes, notably the GPx and TrxR families, support the high-efficiency detoxification of hydrogen peroxide and organic hydroperoxides and sustain thiol redox systems that integrate antioxidant defense with redox signaling [21,22]. Selenium also influences immune competence and inflammatory regulation, in part by shaping the redox environment in which immune cells operate and by modulating pathways that govern inflammatory mediator production and resolution [23]. In parallel, selenium availability affects redox-sensitive gene expression and adaptive responses to oxidative challenge, linking micronutrient status to tissue resilience during stress [21,22].

The production relevance of Se is therefore most apparent in livestock exposed to repeated oxidative challenges driven by management and physiology. High milk yield, rapid growth, and heat stress increase metabolic load and can alter feed intake and nutrient partitioning, collectively raising the demand for antioxidant defenses and efficient redox repair [8,10]. Under these conditions, selenium deficiency can compromise adaptive capacity, weaken immune responses, and reduce productivity, whereas oversupplementation carries a recognized risk because the safety margin between adequate and excessive exposure to this trace element is relatively narrow [20]. Moreover, the biological response to selenium is influenced by the chemical form of supplementation and its bioavailability, with practical implications for nutritional strategies across ruminants and monogastrics and across distinct production stages.

Given the narrow margin between adequacy and toxicity and the strong form- and species-dependence of selenium metabolism, evidence-based selenium management requires integration of redox biology with selenium chemistry and digestive physiology. The review first summarizes dietary Se sources and chemical forms encountered in feeds and supplements, including inorganic oxyanions, selenium-enriched yeast and other selenoamino acid-based products, and emerging forms such as selenium nanoparticles and selenitetriglycerides, with an emphasis on rumen transformations modifying bioavailability. The review then outlines the molecular machinery of Sec incorporation and hierarchical regulation of the selenoproteome, highlighting glutathione peroxidases, thioredoxin reductases, selenoprotein P, and iodothyronine deiodinases as key mediators of peroxide control, immune competence, and thyroid hormone homeostasis. The final sections synthesize experimental and field evidence linking selenium status and supplemental form with clinically relevant outcomes in cattle, pigs, sheep, and goats, including transition-period health, neonatal viability, reproductive performance, and product selenium enrichment. The synthesis emphasizes interactions with vitamin E and other micronutrients, interpretation of biomarkers used in herd monitoring, and risk management in view of the limited safety margin. In line with the predefined scope, the review is limited to mammalian livestock species, namely cattle, pigs, sheep, and goats. Poultry was excluded because avian selenium metabolism, production physiology, and performance outcomes would require a separate comparative framework.

## 2. Characteristics of Selenium

Selenium is an essential trace element with a narrow window between nutritional adequacy and excess, and its biological effects depend less on total intake than on the chemical form that enters the organism and the metabolic routes it follows. Across livestock species, selenium speciation largely determines intestinal availability, conversion into the common metabolic selenium pool, allocation to selenoprotein synthesis, and the rate and chemical form of excretion [24,25]. For practical nutrition, these features translate into marked differences between inorganic salts and organic selenium sources, and into species-specific responses that reflect digestive physiology, particularly the rumen environment in ruminants [26,27,28]. Because Se function intersects with antioxidant defense, endocrine regulation, and immune competence, its status also interacts biologically with other micronutrients that shape redox homeostasis and thyroid metabolism, notably vitamin E, iodine, and iron [29,30].

### 2.1. Occurrence and Chemical Forms

Se is sparsely distributed in the Earth’s crust (approximately 0.00008% by mass), and soil selenium content varies widely across regions, creating pronounced heterogeneity in dietary selenium supply along the trophic chain. In many areas, including much of Europe, low baseline soil selenium limits its accumulation in plants, resulting in forages and crop-derived feeds that provide only marginal selenium unless supplementation is implemented [26,28]. In contrast, distinct geographical zones can present both selenium deficiency and elevated selenium exposure, depending on local geology and soil chemistry, which complicates risk assessment based on region alone and underscores the need for biological monitoring in herds [26].

The biological behavior of Se is determined by its oxidation state and by whether it is present as an inorganic ion, elemental selenium, or an organoselenium compound. As a chalcogen, selenium forms stable compounds across oxidation states −2, +4, and +6, which are reflected in the major inorganic forms encountered in soils and feeds. Inorganic selenium occurs as selenide-containing species and metal selenides, as elemental selenium (Se^0^), and as oxyanions, primarily selenite (SeO_3_^2−^) and selenate (SeO_4_^2−^). Organic selenium encompasses selenium bound to carbon frameworks, including selenoamino acids, methylated selenium metabolites, and selenium incorporated into proteins as Sec within selenoproteins [7,28]. This chemical diversity is biologically important because selenium forms differ in absorption pathways, capacity to generate a reserve pool, and propensity for rapid methylation and elimination when intake exceeds metabolic needs [25,31].

In livestock practice, inorganic selenium is most commonly supplied as mineral salts, typically selenite- or selenate-based additives, which are inexpensive and easy to formulate. Such inorganic species can support selenoprotein synthesis after conversion into reduced selenium intermediates, but they do not provide a large long-term reserve because they are not efficiently incorporated into the general body protein pool. Consequently, a substantial fraction of inorganic Se is directed toward detoxification and elimination, with methylated metabolites appearing in urine and feces; trimethylselenonium (TMeSe^+^) is a well-characterized urinary selenium metabolite in this context [31]. The balance between utilization and excretion is influenced by species, selenium status, and overall diet composition, and it is further modified in ruminants by pre-intestinal transformations within the rumen that reduce the bioavailability of some inorganic forms [26,27].

Beyond traditional inorganic salts and selenium-enriched yeast, newer Se formulations have been explored to modify tissue delivery and widen the safety margin. Selenium nanoparticles (SeNPs) are elemental selenium (Se^0^) preparations engineered at the nanoscale. Experimental studies indicate that SeNPs can elicit strong biological responses and, in some animal models, can maintain or improve antioxidant-related outcomes with lower acute toxicity than conventional selenium compounds. However, biological performance depends strongly on particle size, surface charge, coating, aggregation state, and synthesis method, which limits direct comparison among studies. Mechanistic interpretation also remains incomplete because elemental selenium in SeNP formulations must ultimately be converted to reduced selenium species before selenium can enter selenophosphate-dependent selenocysteine biosynthesis. Current in vivo data support the systemic absorption and metabolic processing of SeNP-derived selenium, but the precise biochemical route from elemental Se0 to the reduced selenium pool remains insufficiently defined. For livestock nutrition, SeNPs should therefore be regarded as promising but not yet mechanistically standardized selenium sources [32,33,34,35].

Organic selenium in feeds is present predominantly as selenomethionine (SeMet) and, to a lesser extent, selenium-containing amino acid derivatives and protein-bound selenium. In plant-derived feed, selenium is incorporated into amino acids through sulfur-related metabolic pathways, leading to SeMet-enriched proteins that enter animal diets. In supplementation, the major organic source is selenium-enriched yeast, in which SeMet is incorporated into yeast proteins and subsequently released during digestion [28]. Organic selenium differs from inorganic forms not only in absorption efficiency but also in metabolic fate. SeMet follows two complementary metabolic routes. It can be nonspecifically incorporated into newly synthesized body proteins in place of methionine, creating a labile selenium reserve, and it can be catabolized to reduced selenium intermediates that support Sec biosynthesis and selenoprotein production [24,28,36]. This dual fate provides a mechanistic basis for the longer retention of organic selenium in tissues and for the observation that organic sources can sustain selenium-dependent functions more effectively when dietary supply fluctuates [28].

Selenitetriglycerides represent a distinct formulation strategy. They are semi-synthetic, lipophilic organoselenium compounds with selenium in the +4 oxidation state, generated by chemical modification of vegetable oil triglycerides and intended to facilitate absorption together with intestinal lipids. Experimental studies in rats have suggested rapid systemic distribution after oral administration, predominant hepatic metabolism, renal excretion, and a substantially lower acute toxicity profile than sodium selenite [37]. In dairy cows, short-term oral supplementation produced a rapid increase in serum selenium. Significant elevations were detectable within 24 h and persisted beyond the supplementation period, without adverse changes in clinical status or selected hematological, hepatic, renal, and metabolic indices at the tested dose [27]. In pregnant sows, repeated prepartum administration increased maternal selenium concentration and glutathione peroxidase activity and enhanced selenium transfer to piglets through colostrum and milk, indicating that selenitetriglycerides can support both maternal selenium status and neonatal selenium supply [38]. Available evidence therefore supports the good biological activity and promising tolerance of selenitetriglycerides in livestock; however, comparative mechanistic data across species and production stages remain limited, and species-specific interpretation should remain cautious, particularly when comparing ruminants with monogastrics [37]. To integrate differences in chemical structure, bioavailability, tissue retention, and practical application among selenium sources, Table 1 summarizes the major selenium forms used in livestock nutrition and their principal biological characteristics.

### 2.2. Mechanisms of Absorption, Transport, and Storage

Selenium absorption occurs primarily in the small intestine, but bioavailability is shaped by selenium speciation, the matrix in which it is delivered, and the digestive physiology of the host. In monogastric species, inorganic selenium oxyanions and organic selenium forms reach the small intestine without extensive pre-intestinal microbial modification, whereas ruminants expose dietary selenium to rumen microflora and a strongly reducing environment before intestinal absorption [24,26]. As a result, apparent selenium absorption is typically lower in ruminants than in nonruminants, with estimates in the veterinary nutrition literature often approximated at 54% in ruminants and 80% in nonruminants, while acknowledging that these values vary with diet composition and selenium form [26]. This species difference is not merely quantitative; it reflects qualitatively different chemistry that can convert absorbable inorganic species into poorly soluble forms.

In the rumen, microbial reduction can transform inorganic selenium salts into elemental selenium or selenides, which have limited solubility and reduced intestinal absorption [39]. This pathway decreases the efficiency of inorganic supplementation and contributes to the common observation that equivalent dietary selenium concentrations can yield different biological responses depending on whether selenium is provided as inorganic salts or as organic sources that better withstand ruminal transformation [4,26,49]. Organic selenium, particularly SeMet within protein matrices, is modified to a lesser extent in the rumen and can be incorporated into microbial protein [40]. This incorporation changes the chemical context in which selenium reaches the small intestine, promoting uptake as part of digested amino acids and increasing the likelihood that selenium enters systemic circulation [26]. Selenitetriglycerides represent a different formulation strategy. They are lipophilic organoselenium compounds generated by triglyceride modification and intended to be absorbed together with intestinal lipids. Available animal studies show rapid increases in circulating selenium after oral administration and support biological activity in calves and periparturient cows [27]. These mechanisms provide a coherent explanation for why organic selenium is often described as more bioavailable in ruminants, even when total selenium intake is similar [40].

In nonruminants such as pigs, both inorganic and organic selenium forms are absorbed in the small intestine without prior rumen-driven transformations, but absorption efficiency and systemic handling still differ by form. Inorganic forms can enter the body through transport pathways shared with related oxyanions and can be rapidly reduced to intermediates that support selenoprotein synthesis, whereas organic forms enter amino acid pools and can be incorporated into body proteins or catabolized to reduced selenium that supports Sec biosynthesis [24,28,49]. Thus, even in monogastrics, absorption does not equate to functional utilization, and selenium form continues to influence whether intake is directed toward immediate selenoprotein synthesis, longer-term storage, or elimination.

After intestinal uptake, selenium circulates in plasma largely bound to proteins. Albumin contributes to the transport of Se species in circulation, whereas selenoprotein P (SeP) is a key selenium transport protein that supports distribution to peripheral tissues and contributes to its homeostasis in the body [21,50]. The liver is central in this element metabolism because it integrates incoming selenium into selenoprotein synthesis and produces SeP for export. Peripheral tissues such as skeletal muscle and kidneys represent major selenium deposition sites, but the form of deposited selenium differs by dietary source. Organic selenium sources that deliver SeMet support incorporation into muscle proteins, which can act as a reserve pool, whereas inorganic selenium more directly supports immediate synthesis needs and can be more rapidly cleared when intake exceeds requirements [24,28]. These features are relevant for interpretation of Se biomarkers because short-term circulating selenium reflects recent intake, whereas tissue and whole-blood measures may better capture longer-term availability shaped by storage and protein turnover [21,50].

Excretion represents a critical component of selenium homeostasis, particularly given the narrow margin between adequate and excessive intake. When its intake exceeds metabolic demand, detoxification pathways methylate selenium, promoting urinary and fecal elimination and reducing cellular retention. Trimethylselenonium is a major methylated selenium metabolite described in urine and is generated by enzymatic methylation processes that become more prominent under higher selenium exposure [31]. From a nutritional perspective, the balance between retention and elimination helps explain why identical supplemental doses can yield different tissue selenium profiles across species and selenium forms, and why risk management must consider both cumulative exposure and form-dependent retention.

### 2.3. Relationships Between Selenium and Other Components of Antioxidant and Hormonal Systems

Selenium biology is tightly interwoven with other micronutrients and antioxidant systems, and these interactions are highly relevant for interpreting Se status and designing supplementation strategies. Among the best characterized relationships is the functional synergy between selenium and vitamin E, a coupling that reflects complementary chemistry across aqueous and lipid compartments of cells. Vitamin E, particularly α-tocopherol, is a major lipophilic chain-breaking antioxidant in membranes and lipoproteins, where it interrupts propagation of lipid peroxyl radicals and thereby limits peroxidative damage to polyunsaturated fatty acids [29]. Selenium-dependent glutathione peroxidases reduce hydrogen peroxide and organic hydroperoxides, including lipid hydroperoxides that arise during membrane oxidation, but the enzyme system has limited access to the hydrophobic core of lipid bilayers. This compartmental separation provides a mechanistic rationale for synergy: α-tocopherol constrains radical chain reactions within membranes, and GPx removes hydroperoxide products that diffuse to accessible interfaces, thereby preventing re-initiation of lipid peroxidation [29,51]. In livestock, combined supplementation with Se and vitamin E has been associated with improved antioxidant indices and stronger immune responses compared with either nutrient alone, supporting the view that the adequacy of both nutrients is required for robust redox buffering during physiological stress [29,52,53]. This interaction is particularly relevant in high-demand windows such as the periparturient period and early postnatal growth, when oxidative load increases and endogenous antioxidant capacity may be insufficient if dietary supply is marginal [29,38].

Selenium and iodine are functionally linked through thyroid hormone synthesis and activation and through protection of the thyroid gland from the oxidative byproducts of hormonogenesis. Iodine is an essential structural component of thyroxine (T4) and triiodothyronine (T3), whereas selenium is required for enzymes that activate and inactivate thyroid hormones and for antioxidant systems that constrain peroxide exposure within the thyroid [30]. Thyroid hormone synthesis requires oxidation of iodide and its incorporation into thyroglobulin, a process catalyzed by thyroid peroxidase (TPO), a heme iron-dependent enzyme that uses hydrogen peroxide (H_2_O_2_) as an oxidant. Because H_2_O_2_ is generated locally and can damage thyroid cells when excessive, efficient peroxide detoxification is essential for gland integrity. GPx activity contributes to H_2_O_2_ removal and thereby supports protection of thyroid tissue during ongoing hormonogenesis [26,30]. Selenium is also required for iodothyronine deiodinases that convert T4 to active T3 or to reverse T3 (rT3), linking selenium status directly to peripheral and tissue-specific thyroid hormone activation [26]. Consequently, Se deficiency can impair thyroid hormone activation even when iodine intake is adequate, whereas iodine deficiency constrains hormone synthesis regardless of selenium status [54]. In iodine-limited settings, selenium supplementation has been reported to support thyroid hormone metabolism and activation by sustaining deiodinase activity, although the magnitude of benefit is context dependent and should be interpreted within the broader nutritional environment [55].

The relationship between selenium and iron is more complex because iron is both essential for antioxidant enzymes and a potential driver of oxidative stress when present in excess. Iron supports oxygen transport through hemoglobin and serves as a cofactor in many enzymes, including catalase, peroxidases, and TPO, thereby intersecting with both redox control and thyroid function [30]. Iron deficiency can reduce thyroid hormone synthesis by limiting TPO activity, potentially amplifying the effects of marginal iodine and selenium supply. Iron adequacy supports cellular oxygen delivery and overall metabolic function, indirectly influencing nutrient utilization [30]. In contrast, iron excess is pro-oxidant and promotes free radical generation through redox cycling reactions, increasing lipid peroxidation risk. Under such conditions, glutathione peroxidase activity can contribute to the mitigation of iron-associated oxidative damage by reducing peroxides that propagate iron-driven lipid oxidation [21,56]. Iron can also modify selenium bioavailability at the level of absorption and early distribution. In pigs, dietary iron concentration and form have been shown to influence Se appearance in portal circulation after feeding, consistent with competition or regulation during intestinal handling and first-pass distribution [57]. Additional evidence suggests antagonism between iron and selenium bioavailability, potentially through formation of less soluble complexes or competition for transport processes. These mechanisms could reduce systemic selenium status in animals exposed to high iron loads [41,58]. Such interactions are practically relevant in settings where feed ingredients or water sources contain elevated iron and where supplementation strategies are designed to maintain selenium-dependent antioxidant function [39]. In such contexts, interpreting selenium biomarkers and clinical indicators without accounting for iron status risks underestimating the nutritional drivers of impaired redox balance [56].

From a veterinary and nutritional perspective, the selenium–vitamin E–iodine–iron axis is best understood as an integrated system that links antioxidant defense, endocrine regulation, and immune competence. Selenium-dependent GPx supports immune cell integrity and peroxide control, vitamin E limits membrane lipid peroxidation, iodine ensures thyroid hormone synthesis, and iron supports oxygen transport and key heme enzymes but can become pro-oxidant when excessive [29,30]. Deficiency or imbalance of any component can increase susceptibility to infectious disease and reduce performance, particularly during stress-sensitive production stages [38,52]. Accordingly, selenium supplementation is most effective when framed within a broader micronutrient strategy rather than treated as an isolated intervention, with attention to form-dependent bioavailability, species physiology, and interacting nutrients that jointly shape redox and endocrine outcomes.

## 3. The Role of Selenium in the Construction of Selenoproteins

### 3.1. Selenium as a Genetically Encoded Catalytic Element

Selenium is distinctive among essential trace elements because many of its core biological functions depend on covalent incorporation into proteins rather than on reversible binding as a cofactor [59,60]. In eukaryotes, Se is integrated into proteins as Sec, the 21st proteinogenic amino acid, which occupies conserved catalytic motifs in redox-active enzymes and related proteins [61,62]. This genetically encoded pathway differs from the nonspecific replacement of methionine by selenomethionine during general protein synthesis, which increases selenium retention but does not target selenium to catalytic active sites [45,63]. The preferential use of Sec in enzyme catalysis reflects physicochemical advantages over cysteine [59,64]. The selenol group of Sec has a lower pKa (approximately 5.2) and higher nucleophilicity than the corresponding thiol, which favors a catalytically reactive state at physiological pH [44,65]. These features support high turnover in oxidoreductases such as glutathione peroxidases and thioredoxin reductases, thereby strengthening peroxide control and intracellular redox homeostasis under fluctuating oxidative load [21,55,61].

### 3.2. Recoding of UGA and the SECIS-Dependent Translation Apparatus

Selenoprotein biosynthesis requires specialized translational recoding that reinterprets an in-frame UGA codon from a stop signal to a Sec insertion codon [64,66]. In mammals, recoding depends on a cis-acting Sec insertion sequence (SECIS) element located in the 3′ untranslated region (3′ UTR) of the selenoprotein mRNA [22,66]. The SECIS element recruits SECIS-binding protein 2 (SBP2) and organizes a dedicated selenoprotein translation complex. This complex interfaces with the ribosome and the Sec-specific elongation factor (eEFSec) to deliver Sec-charged tRNA[Ser]^Sec^ to the ribosomal A site at the UGA codon [59]. Failure of key trans-acting components, particularly SBP2, compromises global selenoprotein synthesis and produces systemic phenotypes consistent with functional selenium deficiency [22]. These observations emphasize that selenium biology depends on a translation-specific decoding pathway rather than on simple substrate availability [22,66].

### 3.3. Sec Biosynthesis on tRNA and Entry of Dietary Selenium into the Functional Pool

Sec is not synthesized as a free amino acid for conventional aminoacylation. Instead, Sec is synthesized directly on its cognate tRNA (tRNA[Ser]^Sec^), a strategy that preserves specificity and prevents nonspecific incorporation into other proteins [60,66]. The pathway begins with seryl-tRNA synthetase (SerRS) charging tRNA[Ser]^Sec^ with serine to form Ser-tRNA[Ser]^Sec^ [22,67]. Phosphoseryl-tRNA kinase (PSTK) converts Ser-tRNA[Ser]^Sec^ to O-phosphoseryl-tRNA[Ser]^Sec^ (Sep-tRNA[Ser]^Sec^), and Sec synthase (SepSecS) then produces Sec-tRNA[Ser]^Sec^ using selenophosphate as the activated selenium donor [64]. Selenophosphate is synthesized from reduced selenium (selenide) and ATP by selenophosphate synthetase 2 (SPS2; gene *SEPHS2*), linking selenium metabolism with selenoprotein synthesis [59,68]. Dietary selenium from organic and inorganic sources must ultimately converge on the reduced selenium pool to sustain selenophosphate generation and Sec biosynthesis [40,44,55,62]. Convergence of Se forms on a shared reduced intermediate provides a mechanistic basis for the form-dependent differences in selenium utilization reported across species and production settings [40,44].

### 3.4. Hierarchical Allocation of Selenium to the Selenoproteome

Selenium availability shapes selenoprotein expression through a hierarchical prioritization mechanism [68,69]. Under selenium limitation, essential selenoproteins are preferentially maintained, whereas some stress-responsive selenoproteins, including GPx1 in many contexts, are downregulated [70]. The hierarchy is influenced in part by isoform-specific modification of tRNA[Ser]^Sec^, because the relative abundance of modified and unmodified tRNA[Ser]^Sec^ species biases translation toward different subsets of selenoprotein mRNAs [67,68]. As a result, selenoprotein synthesis is a regulated homeostatic process that allocates selenium to preserve core redox and metabolic functions during fluctuating supply [69,70]. Prioritization helps explain why standard selenium biomarkers can plateau while specific stress phenotypes remain sensitive to selenium availability [62].

The mammalian selenoproteome comprises a defined set of Sec-containing proteins with diverse subcellular localizations and functional specializations (25 in humans) [59,66,68]. Many selenoproteins are oxidoreductases that support antioxidant defense, thyroid hormone metabolism, and quality control in the endoplasmic reticulum [55,64]. Tissue and compartment distributions reflect local requirements for peroxide control, thiol redox regulation, endocrine homeostasis, protein folding, and organelle integrity [55]. To connect selenium metabolism with the main functional protein products of selenium utilization, Table 2 summarizes representative mammalian selenoproteins involved in redox regulation and cellular homeostasis.

### 3.5. Selenoproteins in Neutralization of Reactive Oxygen and Nitrogen Species

The primary physiological role of selenium is its incorporation into selenoproteins as Sec, which enables high-efficiency redox catalysis rather than the stoichiometric scavenging of oxidants [7,22,25]. Through catalytic turnover, selenoproteins constrain accumulation of ROS and RNS, protecting macromolecules from oxidative modification. They also shape redox-sensitive signaling pathways that coordinate inflammation, metabolism, and cellular responses [10,16,18,85].

Glutathione peroxidases constitute a principal defense against hydrogen peroxide and organic hydroperoxides [59,66,68]. GPx catalysis proceeds through a ping-pong mechanism in which the Sec selenol is oxidized by a peroxide substrate to a selenenic acid intermediate. The active site is then regenerated through a GSH-dependent reaction that produces oxidized glutathione (GSSG) and water or the corresponding alcohol[71]. GPx1 contributes to the rapid detoxification of intracellular H_2_O_2_ in cytosolic and mitochondrial compartments, limiting secondary radical formation and oxidative injury [18,62,86]. GPx4 has a distinct substrate spectrum because GPx4 reduces phospholipid hydroperoxides within membranes, thereby terminating lipid peroxidation chain reactions and suppressing ferroptosis, an iron-dependent form of regulated cell death driven by lipid peroxide accumulation [21,59].

Thioredoxin reductases support a complementary thiol redox system [87,88]. Cytosolic TrxR1 and mitochondrial TrxR2 use NADPH to reduce oxidized thioredoxin, which then provides reducing equivalents to multiple enzymes, including peroxiredoxins that reduce hydrogen peroxide, organic hydroperoxides, and peroxynitrite [2,71,88]. The thioredoxin system also contributes to redox-sensitive transcriptional regulation and to nucleotide metabolism through the support of ribonucleotide reductase activity [60,87]. The C-terminal Sec residue in mammalian TrxRs broadens substrate tolerance and supports rapid kinetics relative to sulfur-dependent homologs [21,22].

Selenoproteins also limit RNS-associated injury, particularly damage driven by peroxynitrite formed from nitric oxide and superoxide [16,18]. GPx enzymes can reduce peroxynitrite and related oxidants, thereby limiting tyrosine nitration and oxidative modification of lipids and proteins [2,3,25,83]. SeP extends antioxidant protection to the extracellular compartment and links systemic selenium status to peripheral tissue delivery, which is relevant to oxidant exposure at vascular and tissue interfaces [2,3].

Selenoproteins additionally contribute to recovery after oxidative stress. MSRB1 reverses oxidation of methionine residues in proteins, restoring the function of oxidatively modified targets and supporting immune cell processes [62,68]. SELENOH and SELENOW are frequently discussed as contributors to redox sensing and stress adaptation programs, although tissue-specific mechanisms continue to be refined [62,81].

### 3.6. Selenoproteins in Membrane Integrity and Mitochondrial Function

#### 3.6.1. Control of Membrane Lipid Peroxidation

Cellular membranes are vulnerable to oxidative injury because polyunsaturated phospholipids support self-propagating lipid peroxidation [5,6,10]. GPx4 is positioned to suppress this process by reducing phospholipid hydroperoxides in situ within lipid bilayers, thereby terminating chain propagation and preserving membrane barrier function [25,44,66]. This activity links selenium availability to resistance against ferroptotic cell death under conditions that promote lipid peroxide accumulation [21,22]. SeP contributes to protection at extracellular and plasma membrane interfaces by supporting selenium delivery and extracellular redox balance [49,70]. Selenium-dependent pathways also intersect with membrane biogenesis because SELENOI catalyzes a key step in phosphatidylethanolamine synthesis. This activity links selenoprotein function to the structural maintenance of cellular and vesicular membranes [59,68].

#### 3.6.2. Mitochondrial Peroxide Control and Apoptosis Susceptibility

Mitochondria are major endogenous sources of ROS due to electron leakage from the respiratory chain, which generates localized oxidant fluxes [18,54,66]. The inner mitochondrial membrane is a prominent target of oxidative damage because it is adjacent to ROS generation sites and is enriched in oxidizable lipids [5,10,81]. Mitochondrial antioxidant defenses include GPx1, GPx4, and TrxR2, which collectively constrain hydrogen peroxide accumulation, reduce lipid hydroperoxides, and maintain the thiol redox state of mitochondrial proteins [22,44,55]. Impairment of these systems promotes loss of mitochondrial membrane potential, cytochrome c release, and caspase activation, linking selenium-dependent redox control to apoptosis susceptibility under oxidative challenge [51,87].

#### 3.6.3. Mitochondrial Content, ER Coupling, and Ca^2+^ Signaling

Beyond immediate peroxide detoxification, several selenoproteins have been linked to the regulation of mitochondrial content and metabolic capacity. SELENOH has been associated with transcriptional programs that support antioxidant defenses and mitochondrial biogenesis. These programs involve regulators such as peroxisome proliferator-activated receptor gamma coactivator 1-alpha (PGC-1α), nuclear respiratory factor 1 (NRF1), and mitochondrial transcription factor A (TFAM), which is consistent with a role in sustaining respiratory competence during stress [81]. SELENOO has been implicated in post-translational regulation through AMP transfer to protein substrates, suggesting an additional route by which mitochondrial selenoproteins may shape stress responses [59,68]. Mitochondrial performance is closely coupled to ER function through mitochondria-associated membranes, which coordinate Ca^2+^ transfer and integrate stress signaling with oxidative phosphorylation. ER-resident selenoproteins contribute to these interfaces by regulating Ca^2+^ handling and protein quality control. SELENON modulates ER and sarcoplasmic reticulum Ca^2+^ homeostasis via interactions with Ca^2+^-handling proteins, including SERCA-associated pathways and ryanodine receptor (RyR)-linked flux [81]. Altered SELENON function has been associated with impaired ER-to-mitochondria Ca^2+^ transfer and reduced bioenergetic output under stress. SELENOW has similarly been linked to Ca^2+^ channel regulation and cellular responses to redox imbalance, supporting the view that the selenoproteome integrates membrane integrity, organelle signaling, and metabolic stability [59,71,82].

## 4. The Importance of Selenium in the Organisms of Farm Animals

### 4.1. Cattle

#### 4.1.1. Impact of Selenium Deficiency on Calves

The importance of selenium for cattle health reflects the central role of selenium-dependent selenoproteins in antioxidant defense and the maintenance of cellular integrity [26,51]. In the neonate, particularly the calf, the most severe clinical manifestation of Se deficiency is nutritional muscular dystrophy, colloquially referred to as white muscle disease, a condition frequently exacerbated by concurrent vitamin E deficiency [26,44,52]. The etiology of such myopathy is rooted in the incapacity of the cellular antioxidant network to neutralize ROS generated during metabolic activity. This failure leads to peroxidation of membrane lipids, inhibition of protein synthesis, and subsequent hyaline degeneration of muscle fibers [40,82]. Such degenerative processes may present in a peracute form, often affecting the myocardium of the neonate and resulting in arrhythmia, tachycardia, and sudden death due to cardiac failure [26,40,44]. Electrocardiographic analyses of affected calves have elucidated distinct abnormalities, including accelerated sinus rhythms, increased P wave amplitude, shorter PR and QT intervals, and altered wave amplitudes—phenomena collectively indicative of early-stage cardiomyopathy and degenerative changes in the heart muscle [40]. Alternatively, a subacute form may manifest, predominantly affecting skeletal muscles, a presentation characterized by stiffness, tremors, and recumbency [40,44,52]. Such clinical signs appear alongside distinct biochemical alterations, notably elevation of serum aspartate aminotransferase, creatine kinase, and lactate dehydrogenase, biochemical evidence occurring concomitantly with depressed GPx activity and reduced serum Se concentrations [40]. Additionally, novel organic formulations such as selenitetriglycerides have demonstrated high bioavailability in neonates, where a single oral administration was sufficient to induce a rapid, three-fold increase in serum selenium concentration and a sustained elevation in glutathione peroxidase activity, further highlighting the efficacy of organic sources in rapidly correcting selenium deficits [83].

#### 4.1.2. Selenoproteins and Redox Homeostasis During High Metabolic Demand

From the transition period to peak lactation, cattle rely on adequate selenium supply for the sustained synthesis of selenoproteins, particularly GPx and TrxR, which support antioxidant defense during high metabolic demand [85,89]. Such maintenance of redox homeostasis becomes critical during the periparturient transition period. This phase is characterized by a dramatic increase in oxygen consumption to support lactogenesis and a simultaneous mobilization of adipose reserves, processes that amplify mitochondrial ROS production and frequently deplete endogenous antioxidant reserves [85]. Investigations indicate that supplementation with organic selenium sources, such as selenium-enriched yeast, hydroxy-selenomethionine (HMSeBA) or selenitetriglycerides, significantly upregulates the activity of serum glutathione peroxidase and thioredoxin reductase compared with inorganic sodium selenite [83,85,86]. For instance, Gong et al. [85] observed that selenium-enriched yeast supplementation enhanced serum selenoprotein P content and thioredoxin reductase activity, thereby reinforcing the systemic capacity to neutralize hydrogen peroxide and lipid hydroperoxides. The superior efficacy of organic selenium forms is largely attributed to altered metabolic fates within the reticulorumen; rumen microorganisms reduce a substantial proportion of dietary inorganic sodium selenite into insoluble elemental selenium, rendering the mineral unavailable for absorption [28,33,38]. In contrast, organic forms like SeMet are incorporated into microbial protein or escape ruminal degradation, facilitating efficient intestinal absorption via amino acid transport mechanisms [33,38]. Consequently, studies demonstrate that cows fed organic selenium exhibit marked improvements in Se status; Koenig and Beauchemin [87] reported an 11% increase in whole blood selenium and a 13% increase in serum selenium in cows fed selenium yeast compared with selenite. Furthermore, Sun et al. [88] established that supplementation with HMSeBA increased plasma selenium by 23% and milk selenium concentrations by approximately 125% relative to inorganic controls, illustrating the profound impact of chemical form on lactational output and antioxidant reserve accumulation.

#### 4.1.3. Reproductive Physiology and Fertility

Reproductive efficiency in cattle is intricately linked to oxidative status, as reproductive tissues are highly susceptible to damage by ROS, which can impair follicular development, steroidogenesis, and embryonic survival [12,54,89]. Selenium deficiency in dairy cattle is associated with an increased incidence of retained fetal membranes (RFMs). This multifactorial pathology is linked to oxidative degeneration of the placental interface and impairment of the neutrophil function required for immune-mediated placental detachment [54,89]. Adequate Se status maintains GPx activity, which protects the placenta from peroxidative damage and facilitates the synthesis of arachidonic acid metabolites essential for uterine involution and placental expulsion [54]. Consequently, Se supplementation has been demonstrated to reduce the incidence of RFM, metritis, and ovarian cysts, thereby decreasing the number of services per conception and shortening the calving-to-conception interval [54,89,90]. Controlled trials have substantiated these benefits. For instance, Khatti et al. [91] observed that combined supplementation of vitamin E and Se, alongside increased energy allowance, significantly ameliorated transition stress, shortening the interval from calving to first estrus by approximately 13 days and increasing the pregnancy rate by nearly 50% compared with unsupplemented controls. Furthermore, herds with adequate Se status exhibit a lower prevalence of metritis and endometritis, likely due to enhanced neutrophil bactericidal capacity and reduced oxidative inflammation within the uterine environment [54,89]. Conversely, the benefits of supplementation appear context-dependent; Cerri et al. [90] reported that replacing inorganic sodium selenite with organic Se yeast in diets that were already adequate in basal Se did not further improve fertilization rates, embryo quality, or uterine health in high-producing dairy cows, suggesting that supranutritional effects may be limited once physiological requirements are met.

Moreover, Se plays a pivotal role in ovarian function and endocrine regulation, specifically in the synthesis and metabolism of progesterone, a hormone critical for the maintenance of pregnancy [89]. Selenium is highly concentrated in healthy preovulatory follicles, where it upregulates the expression of antioxidant enzymes such as GPx1 and superoxide dismutase (SOD) in granulosa cells, thereby protecting the oocyte from oxidative stress during maturation [54]. In vitroapoptosis, buffalo oocyte-derived cell lines have further demonstrated that Se supplementation enhances nuclear maturation rates, reduces apoptosis, and alters the expression of the genes involved in lipid metabolism and steroidogenesis, including the downregulation of Caspase-3 (*Casp3*) and upregulation of *GPX4* and *SOD* [92]. Following ovulation, Se supports corpus luteum function, ensuring adequate progesterone production [93]. Supplementation with organic Se sources has been correlated with elevated plasma progesterone concentrations during late gestation (weeks 29–39), suggesting that selenium may help prevent oxidative damage to luteal tissue and support steroidogenic enzyme activity [93]. Additionally, deficiency in this trace mineral has been linked to increased embryonic mortality during the first month of gestation, indicating that the fertility benefits of Se may arise from preservation of early embryonic viability and the modulation of genes related to maternal recognition of pregnancy, such as interferon-stimulated genes [54].

In the context of male fertility, Se is essential for spermatogenesis and the maintenance of sperm quality, serving functions that are both structural and enzymatic [41]. The mineral is incorporated into the mitochondrial capsule selenoprotein and GPx4, which are critical structural components of the sperm midpiece responsible for maintaining flagellar integrity [66]. Selenium deficiency in bulls is characterized by reduced sperm motility, viability, and increased morphological abnormalities, such as deformed heads or fragile tails, which compromise fertilizing capacity [54]. Although GPx4 enzymatic activity is low in mature sperm, its expression in spermatogonia and spermatids is crucial for protecting developing germ cells and sperm DNA from oxidative fragmentation during spermatogenesis [94]. Supplementation with Se has been shown to improve sperm parameters, including membrane integrity and post-thaw viability, by reducing lipid peroxidation and elevating antioxidant biomarkers such as total antioxidant capacity in seminal plasma [95]. Furthermore, Se may influence the hypothalamic–pituitary–gonadal axis, regulating the secretion of luteinizing hormone (LH) and testosterone, thereby orchestrating the hormonal control of male reproductive physiology [96]. Emerging evidence also suggests that novel delivery systems, such as SeNPs, can protect against the reproductive toxicity induced by environmental toxins like aflatoxin B1, preserving testicular architecture and reducing DNA fragmentation in bulls [54,97,98]. Thus, adequate Se status is critical for optimizing reproductive efficiency in both male and female cattle, influencing outcomes from gametogenesis through to parturition [54].

#### 4.1.4. Immunological Competence and Passive Transfer

The immunomodulatory properties of selenium are critical for bolstering both innate and adaptive immune responses, particularly during the immunosuppressive transition period when the dairy cow is most vulnerable to metabolic and infectious challenges [85]. In the context of innate immunity, neutrophils rely heavily on the cytosolic activity of selenium-dependent glutathione peroxidase to protect themselves from the cytotoxic effects of superoxide radicals. These radicals are generated during the respiratory burst used to kill ingested pathogens [54,85]. Investigations into the functional capacity of leukocytes have demonstrated that Se deficiency impairs neutrophil migration (chemotaxis) and bactericidal activity, rendering cows more susceptible to infectious diseases such as mastitis and metritis [54,85,99]. For instance, Malbe et al. [99] have observed that in Se-deficient herds, supplementation with either selenium-enriched yeast or sodium selenite significantly improved udder health; specifically, the percentage of quarters harboring mastitis pathogens dropped from 22.9% to 13.0% in the Se-yeast group and from 18.4% to 7.4% in the selenite group during the supplementation period. Furthermore, chemiluminescence assays revealed that neutrophils from Se-supplemented cows exhibited a modified oxidative burst profile, characterized by a more rapid and sustained peak (skewed to the left), indicating a more efficient generation of ROS for pathogen elimination without compromising the viability of the phagocyte.

Beyond cellular defense mechanisms, Se supplementation exerts a profound influence on the production of cytokines and immunoglobulins, which are pivotal for the orchestration of the adaptive immune response. Gong et al. [100] demonstrated that cows supplemented with selenium-enriched yeast exhibited significantly elevated serum concentrations of interleukin-1 (IL-1) and immunoglobulin A (IgA) compared with those receiving inorganic sodium selenite, suggesting that organic sources may more effectively stimulate specific arms of the immune system. Such modulation of the cytokine profile facilitates the differentiation of macrophages toward an anti-inflammatory phenotype, thereby promoting the timely resolution of inflammation and preventing tissue damage associated with chronic oxidative stress [54]. This enhanced immunological status translates to tangible clinical benefits; herds with optimized Se status frequently present with reduced somatic cell counts (SCCs) and a lower prevalence of environmental mastitis, particularly infections caused by *Escherichia coli*, where the rapidity of neutrophil recruitment is the primary determinant of infection outcome [85,100].

A pivotal aspect of Se immunobiology in cattle is its role in the passive transfer of immunity from the dam to the neonate, a process essential for calf survival given the synepitheliochorial nature of the bovine placenta, which prevents in utero immunoglobulin transfer. Supranutritional supplementation of the dam during late gestation has been empirically shown to enhance the efficiency of immunoglobulin G (IgG) absorption by the newborn calf [101,102]. In a seminal study, Hall et al. [101] reported that calves born to cows supplemented with selenium-enriched yeast (105 mg Se/week) during the last 8 weeks of gestation exhibited a 62% higher apparent efficiency of IgG absorption compared with controls, resulting in significantly higher serum IgG concentrations at 48 h of age. The underlying mechanism is postulated to involve Se-mediated regulation of intestinal epithelial cell turnover or the direct stimulation of enterocyte pinocytosis prior to gut closure [101,102]. Furthermore, Żarczyńska et al. [53] demonstrated that parenteral administration of a Se and vitamin E supplement (30 mL single dose) to pregnant cows 10 days prior to the expected parturition date significantly increased serum Se concentrations in the offspring and promoted the passive transfer of immunity, as evidenced by elevated serum IgG levels in 24 h-old calves compared with unsupplemented controls [53]. Collectively, these data indicate that selenium functions not only as an antioxidant but also as a modulator of immunological competence, essential for ensuring the survival and health of both the periparturient cow and the neonate.

#### 4.1.5. Lactational Performance and Milk Composition

While the influence of Se on milk yield remains debated, with some studies reporting no significant increase in volume and others observing marginal gains under specific conditions such as heat stress, its effect on milk composition, specifically Se concentration, is unequivocal. Comprehensive investigations into the dietary selenium source, specifically comparing organic forms (such as selenium-enriched yeast or HMSeBA) with inorganic salts (sodium selenite or selenate) have yielded divergent results regarding overall lactational performance. A substantial body of literature indicates that neither the source nor the quantity of selenium supplementation significantly alters dry matter intake (DMI), milk yield, or the protein and lactose content of milk in cattle under thermoneutral conditions or adequate basal selenium status [100,103,104,105]. For instance, Koenig and Beauchemin [103] have reported that supplementing total mixed rations (TMRs) with 0.3 mg/kg Se as either selenium-enriched yeast or sodium selenite for 60 days prepartum through 60 days in milk resulted in no detectable differences in milk production or major milk solids. Similarly, Gong et al. [100] observed that milk yield and composition (fat, protein, lactose) remained unaffected by selenium source in mid-lactation cows, reinforcing the view that selenium’s primary role is not as a direct driver of lactogenesis.

However, responses may vary depending on experimental contexts. Phipps et al. [46] observed a positive linear effect on milk yield as the inclusion rate of selenium-enriched yeast increased from 0.30 to 0.45 mg Se/kg dry matter (DM), although the source of selenium (yeast vs. selenite) did not independently alter yield. Furthermore, the metabolic state of the animal appears to modulate responsiveness; Sun et al. [106] demonstrated that, under oxidative stress induced by heat stress, cows supplemented with HMSeBA tended to exhibit higher milk yields (approximately 9.3%) than cows receiving inorganic sodium selenite. This response may reflect enhanced antioxidant capacity that spares metabolic energy for lactation. Regarding milk fat synthesis, results are conflicting but suggest a bioactive role for organic selenium forms. Oltramari et al. [104] have reported a significantly higher milk fat percentage (4.15% vs. 3.96%) in cows receiving organic selenium yeast compared with inorganic sources, a finding corroborated by Sun et al. [105], who noted a linear increase in milk fat yield and 4% fat-corrected milk (FCM) with increasing doses of HMSeBA. Conversely, under severe heat stress, HMSeBA supplementation was associated with a reduction in milk fat percentage compared with inorganic controls [106], highlighting the complex interaction between redox status, selenium form, and lipid metabolism.

In contrast to the variable effects on yield, the impact of selenium supplementation on the selenium concentration in milk is profound and consistent. A meta-analysis of 42 studies concluded that oral supplementation with organic selenium increases milk selenium concentrations significantly more than inorganic forms [107]. This superiority of organic sources is attributed to the nonspecific incorporation of SeMet into milk proteins, particularly casein, in place of methionine [46,108,109]. Givens et al. [108] demonstrated that, while sodium selenite supplementation increased milk Se concentration by only 4 µg/L, an equivalent dose of selenium-enriched yeast resulted in an increase of 65 µg/L. Phipps et al. [46] further elucidated this mechanism, showing that replacing sodium selenite with selenium-enriched yeast increased the SeMet concentration in milk from 36 to 111 ng Se/g dried sample, effectively tripling the SeMet fraction [46]. Walker et al. [109] have established that Se secretion in milk increases linearly with intake, accounting for approximately 17% of total selenium intake in yeast-supplemented cows. Additionally, novel organic sources such as HMSeBA have been shown to elevate milk selenium concentrations by up to 125% compared with sodium selenite, exhibiting a higher milk-to-plasma selenium transfer efficiency [105]. This enrichment extends to colostrum, where maternal organic selenium supplementation significantly enhances colostral selenium reserves available to the neonate, often exceeding concentrations achieved by inorganic supplementation by over 40% [101,103]. Interestingly, recent speciation data suggest that, while SeMet drives total milk selenium, elevated colostral selenium in HMSeBA-supplemented heifers may result from increased Sec rather than SeMet, suggesting differential transport mechanisms during colostrogenesis [45]. Finally, organic selenium has been linked to improved udder health, as evidenced by reduced SCC in cows supplemented with selenium yeast compared with inorganic forms [104], likely due to enhanced neutrophil function and antioxidant defense within the mammary gland. To consolidate evidence in cattle across antioxidant status, reproduction, immune function, passive transfer, and milk-related outcomes, Table 3 summarizes the major reported physiological and production effects of the principal selenium forms used in cattle.

### 4.2. Pigs

#### 4.2.1. Importance of Selenium for Porcine Health and Immunity in the Context of Oxidative Stress

##### Sows and Neonatal Piglets

Contemporary literature describes selenium as a core component of the porcine antioxidant defense system, essential across all life stages and supporting normal growth, reproduction, and health. Selenium influences multiple stages of reproduction in pigs, including ovarian follicle maturation, embryo implantation, and placental and fetal development. These effects are attributable, in part, to the role of selenium in antioxidant systems that regulate the balance between oxidants and antioxidants required for normal pregnancy [36]. This trace element is particularly important in sow nutrition during gestation and lactation because selenium is efficiently transferred to the fetus and to suckling piglets via the placenta and mammary gland, promoting development of antioxidant capacity from the first days of life [11]. Studies conducted by Świerczyński et al. [38] have demonstrated that oral supplementation of selenitetriglycerides in sows, initiated two weeks before parturition and administered at a dose of 0.5 mg Se/kg body weight, resulted in a significant increase in selenium concentrations both in periparturient sows and in their offspring. Selenium levels in piglets born to supplemented sows were nearly twofold higher than those observed in piglets from unsupplemented sows. Moreover, selenitetriglyceride supplementation induced a rapid and pronounced increase in glutathione peroxidase activity in sows, which was maintained postpartum, thereby providing effective protection against the oxidative stress associated with the high metabolic load during the periparturient period.

Parturition and the immediate postnatal period involve major cardiorespiratory adaptations associated with an increase in tissue partial pressure of oxygen, resulting in physiological oxidative stress. Consequently, neonates experience a high oxidative burden and show heightened susceptibility to free radical-mediated oxidative injury [36]. Yin et al. [112] have reported that immaturity of the antioxidant system in newborn piglets further exacerbates parturition-associated oxidative stress. When sows experience pronounced periparturient oxidative stress (e.g., due to antioxidant deficiency), this burden can be transmitted to the offspring, increasing oxidative load in piglets after birth [84]. Adequate selenium supply in sows can attenuate this phenomenon because improved maternal antioxidant status indirectly protects piglets. Piglets born to sows receiving selenium supplementation have been shown to exhibit higher antioxidant enzyme activity and improved immune parameters in the first days of life. In addition, increased immunoglobulin concentrations (IgG and IgA) have been reported in sows supplemented with organic selenium, which translates into more effective passive immunity transferred to piglets via colostrum [113].

The sow mammary gland is particularly susceptible to oxidative stress during the periparturient period and lactation, which can predispose animals to mastitis-metritis-agalactia (MMA) and postpartum dysgalactia syndrome (PDS). Selenium supports protection of mammary epithelial cells from oxidative injury and excessive inflammatory activation. Adequate selenium supply may modulate immune responses and pro-inflammatory cytokine production and improve the antioxidant status of colostrum and milk, thereby supporting prevention of mammary inflammation in sows [6,24,36]. Selenium is also important for maintaining boar fertility and semen quality because, as a component of antioxidant selenoproteins, it protects spermatozoa from oxidative damage, limits lipid peroxidation of cell membranes, and supports structural stability of the acrosome and flagellum. In this way, selenium contributes to normal sperm motility, viability, and morphological integrity, supporting fertilizing capacity [26,36].

##### Weaned Piglets

Weaning is a critical stage in pig production. As noted above, the antioxidant and immune systems in young piglets are not fully mature, increasing susceptibility to homeostatic disruption and infections. Abrupt weaning, typical of commercial production systems, disturbs physiological equilibrium, intensifies oxidative stress, and promotes cellular injury. Importantly, weaned piglets are also exposed to multiple external stressors, including social, nutritional, environmental, and pathogen-related factors that further promote oxidative stress and inflammation [11]. Through its antioxidant activity, selenium can help prevent damage to cellular membranes and tissue structures during weaning stress, supporting improved growth and reducing mortality in piglets [114]. Weaning also activates inflammatory responses and increases synthesis of pro-inflammatory cytokines, representing an important component of the stress response [115]. Nutritional strategies aimed at mitigating intestinal inflammation effectively support growth and overall health in weaned piglets [116].

Multiple newer selenium sources, particularly organic forms, have been reported to exert anti-inflammatory effects in weaned piglets. Liu et al. [117] demonstrated that supplementation with selenium-enriched yeast alleviated adverse effects of oxidative stress in weaned piglets, attenuating oxidative stress-associated growth suppression, increasing glutathione peroxidase activity and other antioxidant enzyme activities, supporting immune function, and reducing pro-inflammatory cytokine levels.

The gastrointestinal tract is a particularly vulnerable system in piglets during the post-weaning period. The intestinal immune system is considered to reach adult-level competence only by the seventh week of life [118]. Stressors such as separation from the dam, the transition from liquid to solid feed, and environmental change influence immune responses and contribute to health disturbances, characterized by reduced feed intake, decreased growth rate, and increased susceptibility to pathogens within the first 24–48 h after weaning. As a consequence, digestive and absorptive capacity are impaired. In parallel, insufficient secretion of digestive enzymes limits the ability of piglets to digest solid feed, contributing to disruption of the intestinal barrier, including damage to tight junctions, reduced mucin secretion, increased intestinal permeability, and dysregulation of the intestinal microbiota [119].

The impact of weaning stress is not limited to barrier function and microbiome but is also reflected in intestinal immunity and the intestinal redox state in weaned piglets compared with pre-weaning piglets. Intensified oxidation processes during this period lead to excessive release of reactive oxygen species, which can modify cellular proteins and contribute to increased expression of pro-inflammatory cytokines, adversely affecting expression of tight junction proteins and ultimately further increasing intestinal permeability. It has also been suggested that the effects of weaning stress on immune function, the intestinal barrier, and the nervous system in early-weaned piglets may persist into adulthood [120]. To minimize weaning-associated stress, multiple nutritional strategies have been implemented, including optimization of dietary protein and energy content and the use of post-weaning feed additives (e.g., probiotics and prebiotics). Considerable attention has been directed toward the protective effects of selenium on the gastrointestinal system in the post-weaning period [61].

Administration of selenium-enriched mannan oligosaccharides (MOS) has been reported to alleviate diarrhea induced by enterotoxigenic *Escherichia coli* (ETEC) in weaned piglets and strengthen antioxidant and immune functions. With respect to intestinal integrity, MOSS supplementation reduced serum diamine oxidase, D-lactate, and endotoxin concentrations and increased expression of zonula occludens-1 (ZO-1) in both the jejunum and ileum. A reduction in crypt depth (CD) in the jejunum and ileum was also observed, accompanied by an increased villus height-to-crypt depth ratio (VH/CD) in both intestinal segments [121]. Studies by Xiong et al. [113] have demonstrated that supplementation of selenium-enriched yeast to sows during late gestation and lactation improved piglet growth performance during the first week of life, increased selenium status, enhanced antioxidant capacity, and promoted immunoglobulin transfer. According to the authors, these mechanisms contributed to strengthening the small intestinal barrier function and activating the Nrf2/Keap1 signaling pathway in the offspring.

A beneficial effect of selenium-enriched *Cardamine violifolia* (Brassicaceae), a plant with strong selenium-accumulating capacity, on intestinal health in weaned piglets has been reported by Wang et al. [122]. Dietary inclusion of selenium-enriched *C. violifolia* improved jejunal morphology, intestinal enzyme activity, and barrier function in piglets challenged with lipopolysaccharide (LPS). These effects included increased villus height (VH), higher VH/CD, increased lactase and maltase activities, and increased expression of tight junction proteins such as occludin and ZO-1 in the jejunum. The same study also reported improved antioxidant and immune indices, including increased total antioxidant capacity and SOD activity and decreased interleukin-6 (IL-6) concentrations. In experimental models in gilts, supplementation with an organic selenium form, such as HMSeBA, at a dose of 0.3 mg Se/kg feed resulted in a marked increase in intestinal immunoglobulin A (sIgA) levels in the jejunum and ileum compared with animals receiving an inorganic selenium source (sodium selenite) at the same dose, indicating a more effective support of local immune responses and intestinal mucosal barrier integrity by organic selenium forms [123].

##### Growing-Finishing Pigs

Although immune and antioxidant systems are more stable later in growth and during finishing than in early life, appropriate selenium supply at this stage remains relevant to health and production outcomes. Growing-finishing pigs may experience oxidative stress due to rapid growth, high-energy diets, and adverse environmental factors (e.g., high ambient temperature and stocking density) or transport stress. High antioxidant enzyme activity supported by selenium helps protect tissues, particularly muscle, from oxidative injury. Adequate selenium status in finishing pigs has also been associated with improved meat quality and extended shelf life [86]. However, evidence regarding the effects of selenium supplementation on growth rate is inconsistent, and most reports have not identified clear effects of selenium source or dietary dose on production performance [86,124].

#### 4.2.2. Comparison of the Effectiveness of Various Forms of Supplementation

Many studies comparing the effectiveness of organic and inorganic selenium in pigs indicate the advantage of organic forms. The advantages of using organic forms of selenium in sow nutrition result from the ability to store selenium in tissues, primarily in muscles, and utilize these reserves under conditions of antioxidant stress, as well as from more efficient Se transport from the mother to the fetus and newborns via the placenta, colostrum, and milk [36,125]. As reported by Surai and Fisinin [36], compared with inorganic forms, the use of organic Se in sow diets provides higher Se concentrations and improved antioxidant status in weaned piglets, and also improves thyroid metabolism and increases the activity of key digestive enzymes in the piglets’ pancreas during this period. Furthermore, different organic forms of this element are characterized by varying effectiveness in terms of bioavailability and the efficiency of selenium supply. A study by Świerczyński et al. [38] showed that the serum selenium concentration in piglets on the third day of life, from sows supplemented four times with selenitetriglycerides (15, 10, 5, and 3 days before farrowing), was higher than the values reported by Falk et al. [125] in piglets on the fifth day of life, whose mothers received selenium in feed in the form of sodium selenate or SeMet for a month before farrowing. In their study, Doan et al. [126] showed that supplementation of 0.3 mg/kg of selenium in the form of selenium-enriched yeast stimulates systemic antioxidant capacity and increases resistance to oxidative stress and inflammation induced by intraperitoneal injection of diquat in weaned piglets. The authors further noted that neither sodium selenite nor selenium combined with soy protein demonstrated antioxidant activity comparable to selenium-enriched yeast in combating diquat-induced oxidative stress in these animals. A study by Rao et al. [127], in which individual groups of piglets received selenium after weaning in the form of sodium selenite, selenium-enriched yeast, and HMSeBA, showed that the Se source had no effect on increased glutathione peroxidase activity. Similar conclusions were reached earlier by Mahan et al. [128], who observed no differences in serum GPx activity between fattening pigs fed diets supplemented with sodium selenite or selenium-enriched yeast at a dose of 0.3 mg/kg. A meta-analysis conducted by Zhou et al. [129] to determine the effect of different selenium sources on sow reproductive performance and piglet development showed that sows receiving organic forms of selenium had 6.4% higher GPx activity compared with sows receiving inorganic Se in their diet. Based on the same meta-analysis, the authors concluded that the administration of organic selenium to sows and piglets improved the antioxidant activity of sows and piglets during pregnancy, lactation, and piglet growth, which is of great importance for their health, production, and reproductive performance. To consolidate evidence in pigs across gestation, lactation, neonatal development, weaning, and growth, Table 4 summarizes the major reported physiological and production effects of the principal selenium forms used in pigs.

### 4.3. Small Ruminants

#### 4.3.1. Etiology and Prevention of Nutritional Muscular Dystrophy

Nutritional muscular dystrophy (NMD; white muscle disease) is a deficiency-associated myopathy in which inadequate antioxidant capacity permits oxidative injury to skeletal and/or cardiac muscle. Selenium deficiency, often accompanied by insufficient vitamin E, impairs peroxide detoxification by Se-dependent antioxidant enzymes and favors accumulation of reactive oxygen species and lipid hydroperoxides. Oxidative injury and lipid peroxidation can progress to clinically relevant cardiomyopathy and/or myopathy, consistent with the broader spectrum of oxidative stress-associated disorders reported in ruminants [24,111,130,131].

Small ruminants are especially vulnerable to Se deficiency syndromes because rumen microorganisms can convert dietary Se (particularly inorganic Se salts) into poorly soluble, less absorbable forms. As previously noted, ruminal microbial activity reduces Se bioavailability in ruminants (approximately 54%) compared with nonruminants (approximately 80%), and inorganic Se sources in powder form may be more readily reduced to insoluble elemental Se [40,132]. Encapsulation of inorganic Se salts has been proposed as an approach to reduce ruminal losses by delaying microbial transformation until the small intestine [132]. Monitoring and case workups for Se-responsive oxidative myopathies commonly use functional indices of Se-dependent antioxidant capacity and markers of lipid peroxidation. Whole-blood glutathione peroxidase activity is widely used as a functional biomarker of longer-term Se status, whereas malondialdehyde (MDA) is used as a marker of lipid peroxidation and oxidative stress [45]. In lambs, acute Se deficiency has been associated with cardiovascular disease accompanied by alterations in oxidative stress-related biomarkers [133].

Prevention of NMD in lambs and kids relies on maintaining adequate maternal selenium supply during late gestation and early lactation, as maternal status influences concentrations in blood, colostrum, and milk, and the element can reach the developing fetus via placental transfer. In late-pregnant ewes, supplementation with an organic form increased Se concentrations in placenta, serum, and colostrum compared with supplemented animals, supporting improved neonatal Se availability at birth and during early lactation [134]. In heavily pregnant and lactating ewes, both organic (selenium-enriched yeast) and inorganic selenium (sodium selenite) supplementation increased blood Se compared with controls [135]. Selenium dose selection should reflect physiological stage, baseline status, and chemical form, given the narrow safety margin of this element [42,136]. The chemical form of Se is a practical determinant of preventive efficacy and safety. Organic sources (e.g., SeMet and selenium yeast) generally show higher absorption, higher bioavailability, and greater tissue storage and are often described as less toxic than inorganic sources, whereas soluble inorganic salts can be comparatively more toxic [43,110,137,138]. Selenium yeast has been reported to have bioavailability of approximately 120–200% relative to inorganic sources [139]. Selenium nanoparticles (nano-Se) have been proposed as an alternative selenium source because smaller particle size and increased surface area can increase mucosal permeability and intestinal absorption, and nano-Se has been described as having lower toxicity than conventional Se sources; however, responses remain dependent on dose and particle characteristics [47,48,131].

#### 4.3.2. Modulation of Immunological Competence and Resilience to Infection

Selenium contributes to immune competence by supporting antioxidant protection within immune cells and by enabling continuous synthesis of Se-dependent enzymes that neutralize ROS and lipid peroxides. Oxidative stress has been linked to inflammatory activation and to multiple disorders in ruminants, including mastitis and myopathic syndromes; therefore, adequate Se supply supports immune defense indirectly by limiting oxidative injury and directly through immunomodulatory effects [111,130,131]. Evidence summarized across ruminant models indicates that Se influences both humoral and cellular immunity through modulation of cytokine secretion and through effects on lymphocyte proliferation and differentiation. Selenium deficiency has been associated with impaired immune defense, whereas adequate Se supply has been associated with improved immune responsiveness [140,141,142]. In lambs, dietary organic Se improved immune response alongside hepatic antioxidant status and selenoprotein-related gene expression compared with unsupplemented controls [143].

Selenium status may also contribute to resilience under heat stress, a condition that increases oxidative stress and can suppress immune function. In heat-stressed sheep, Se (often combined with vitamin E) has been associated with improved antioxidant status and improved production-related outcomes compared with unsupplemented animals [144,145,146]. Because heat stress can also disrupt gut barrier integrity and microbiota composition, antioxidant-oriented supplementation has been discussed as one component of broader mitigation strategies [147]. In the mammary gland, Se supplementation has been linked to markers of udder health in dairy goats. In lactating goats, selenium-enriched yeast increased milk yield parameters and decreased somatic cell count compared with sodium selenite, supporting a practical advantage of organic form for reducing inflammatory indices in milk [148]. Across ruminants, Se status has also been discussed as a factor associated with subclinical mastitis risk and inflammatory responses [131,141].

#### 4.3.3. Reproductive Physiology and Fertility

Reproductive performance in sheep and goats depends on adequate trace mineral supply, and Se requirements increase as gestation advances. Late pregnancy is a period when supplementation may be necessary to match rising maternal–fetal demands, particularly in Se-deficient environments [149]. Inadequate selenium supply in ewes and does has been associated with reduced fertility, abortion, and retained placenta, with oxidative stress implicated as a contributing factor in periparturient reproductive disorders [135,140]. In heavily pregnant and lactating ewes, both organic (selenium-enriched yeast) and inorganic Se (sodium selenite) supplementation increased blood Se concentrations compared with controls [135]. In goats, slow-release supplementation administered during late pregnancy increased Se concentrations and glutathione peroxidase activity in does and their kids and was associated with improved milk production parameters [150].

Male fertility is sensitive to Se status because spermatozoa are vulnerable to lipid peroxidation. In bucks, Se supplementation has been associated with improved testicular characteristics, semen quality, and reproductive hormones such as luteinizing hormone and testosterone [151,152,153]. In rams, injectable Se combined with vitamin E increased ejaculate volume and improved semen-related outcomes compared with controls [154,155]. During semen cryopreservation, in vitro supplementation of extenders with nano-Se improved post-thaw sperm quality and reduced lipid peroxidation compared with unsupplemented extenders [156]. Proposed explanations include enhanced spermatogenesis via antioxidant protection and associations with glutathione peroxidase activity in testicular tissue, as well as direct testicular effects and indirect effects mediated by anterior pituitary hormones [157].

#### 4.3.4. Synergism with Vitamin E and Antioxidant Stability of Milk

In small dairy ruminants, antioxidant pathways are relevant for mammary gland health and for milk oxidative stability because antioxidant protection reduces peroxidation of milk lipids and supports maintenance of milk quality indices under oxidative challenge [158,159]. In lactating ewes, combined oral supplementation with α-tocopherol and sodium selenite altered milk lipid composition by increasing long-chain fatty acids and decreasing short- and medium-chain fatty acids compared with controls; the study also evaluated milk oxidative stability under these supplementation conditions [158]. In dairy goats, supplementation strategies that combined organic Se (selenium-enriched yeast) with vitamin E increased the proportion of long-chain and unsaturated fatty acids in milk compared with unsupplemented animals [159].

The Se source influences transfer into milk and can affect udder inflammation indices. Organic Se supplementation in lactating goats increased milk yield and reduced SCC compared with sodium selenite [148]. In dairy goats, organic and inorganic Se both increased milk yield and Se concentration in milk and tissues, with the organic form reported as more effective than the inorganic form at comparable inclusion rates in the cited study [160]. Slow-release selenium boluses administered during late pregnancy increased milk Se concentration and improved milk production parameters in goats, supporting sustained micronutrient supply across the periparturient period [150].

## 5. Conclusions and Future Perspectives

Selenium is a central determinant of antioxidant capacity in cattle, pigs, and small ruminants because Se is incorporated as Sec into selenoproteins, including glutathione peroxidases and thioredoxin reductases, which detoxify peroxides and maintain thiol redox homeostasis. Across production systems, adequate selenium supply supports tissue resilience during periods of increased oxidant pressure (the periparturient period, heat stress, and weaning), with downstream relevance to immune competence, reproductive performance, and product quality. At the same time, selenium nutrition requires careful management because this trace element has a narrow margin between adequacy and excess, and dietary form governs absorption, tissue retention, entry into the functional Se pool for Sec biosynthesis, and elimination via methylated metabolites when intake exceeds metabolic demand.

The biological response to Se depends less on total intake than on chemical speciation and host digestive physiology. In ruminants, rumen microorganisms can reduce inorganic Se salts to poorly soluble forms, decreasing bioavailability relative to nonruminants and contributing to form-specific differences in systemic status. Organic sources that deliver SeMet, such as selenium-enriched yeast, can better withstand ruminal transformations and enter amino acid pools, enabling nonspecific incorporation into body proteins and creation of a mobilizable reserve that supports selenoprotein synthesis during fluctuating supply. Consistent with these mechanisms, organic Se sources, including HMSeBA, frequently increase Se concentrations in blood and milk more effectively than inorganic salts and can enhance antioxidant indices during high metabolic demand and oxidative stress-inducing conditions. In monogastrics, Se form remains biologically relevant because different sources differ in tissue deposition, maternal–offspring transfer, and the extent to which selenium is routed into storage versus immediate utilization for selenoprotein production.

### 5.1. Research Priorities: Connecting Novel Se Sources with Tissue Allocation, Selenoprotein Biology, and Functional Biomarkers

Newer Se formulations, including SeNPs and selenitetriglycerides, are being explored to modify absorption kinetics and improve practical tolerance compared with conventional ionic salts. However, the critical research question is whether these formulations consistently supply Se to the reduced selenium pool that supports selenophosphate generation and Sec biosynthesis, thereby increasing functional selenoprotein synthesis rather than only increasing nonspecific tissue Se accumulation. Evidence summarized in this review indicates that selenitetriglycerides, a lipophilic Se source intended to be absorbed with intestinal lipids, can rapidly increase blood Se status and GPx activity after oral administration in calves and can improve its transfer to offspring, supporting the feasibility of this delivery strategy during physiologically demanding periods. In parallel, SeNPs warrant cautious evaluation because reported outcomes depend on particle characteristics, and robust standardization is required to interpret bioavailability and safety across studies and species. A central unresolved issue is the biochemical route by which elemental selenium in nanoparticle preparations is converted to reduced selenium for selenophosphate generation and subsequent Sec biosynthesis in vivo.

Progress in this area will require biomarker strategies that can distinguish functional sufficiency from simple exposure. Conventional indices (circulating Se concentration and GPx activity) remain informative for status assessment, but interpretation must account for hierarchical allocation of Se to the selenoproteome, which can produce plateaus in some biomarkers while stress responsiveness remains sensitive to Se availability. Speciation-focused approaches that quantify the chemical forms of Se in relevant matrices (e.g., SeMet- versus Sec-associated Se in milk and colostrum) provide additional resolution for evaluating how supplementation form shifts Se partitioning between nonspecific protein incorporation and selenoprotein-directed utilization. Coupling these measures with markers of oxidative injury (e.g., malondialdehyde) can further connect Se form and status to biologically meaningful redox outcomes.

Future work should include tissue-level characterization of the selenome in organs central to metabolic remodeling and reproductive health under oxidative stress in dairy cows, sows, and their offspring. Adipose mobilization and hepatic adaptation are prominent features of the periparturient period, and postpartum uterine disease in dairy cows and sows often develops in association with compromised antioxidant capacity and immune function. An operational definition of the adipose, hepatic, and uterine selenome that comprises tissue Se concentration, Se speciation, and expression patterns of key selenoproteins mediating peroxide detoxification (GPx), thiol redox control (TrxR), and cellular stress handling would enable mechanistic testing of whether selenitetriglyceride supplementation shifts organ-level Se allocation and selenoprotein deployment in directions that correspond to systemic antioxidant indices and clinically relevant reproductive and inflammatory phenotypes summarized in the present review. Complementary experimental contrasts spanning Se deficiency, Se adequacy, and supplementation with chemically distinct Se forms would also support evaluation of epigenomic responses to Se status, including DNA methylation, histone modifications, and chromatin accessibility in adipose tissue, liver, and uterus, thereby linking Se-dependent regulatory remodeling to redox and immune pathways and tissue-specific phenotypes across the periparturient period.

### 5.2. Practical Implications for Veterinarians and Nutritional Advisors

For veterinary practitioners and nutritional consultants, Se management should move beyond a deficiency-only framework and incorporate form-specific bioavailability, physiological stage, and the narrow safety margin of Se. In ruminants, the tendency for ruminal transformations to reduce inorganic Se availability supports the practical advantage of organic sources when the goal is to sustain systemic status across stress-sensitive windows or to enhance Se transfer into milk and colostrum. In pigs, maternal selenium form is a tractable determinant of neonatal Se status and early-life antioxidant capacity, and post-weaning strategies that target intestinal barrier function and inflammatory tone can incorporate organic Se sources as part of broader nutritional mitigation strategies. Across species, monitoring should incorporate functional readouts of antioxidant capacity (GPx activity) and, where feasible, complementary markers that reflect oxidative injury and Se partitioning. Finally, implementation of novel delivery systems (including SeNPs and selenitetriglycerides) should proceed alongside careful evaluation of tissue distribution, consistency of biomarker responses, and demonstrable incorporation into selenoprotein-dependent antioxidant pathways to ensure that improved bioavailability translates into durable physiological benefit without increasing the risk associated with excess Se exposure.

## Figures and Tables

**Table 1:** Major selenium forms used in livestock nutrition and their biological characteristics.

Selenium Form	Chemical Class	Main Route/Metabolic Behavior	Storage Potential	Main Practical Advantages	Main Limitations/Cautions	References
Sodium selenite	Inorganic oxyanion (Se IV)	Rapidly enters the common reduced Se pool after reduction; in ruminants may be reduced in the rumen to poorly soluble forms	Low	Widely used, inexpensive, supports selenoprotein synthesis	Lower bioavailability in ruminants; limited tissue storage; comparatively higher toxicity than organic forms	[24,39,40,41,42,43]
Sodium selenate	Inorganic oxyanion (Se VI)	Absorbed as an oxyanion; enters selenium metabolism after reduction	Low	Effective inorganic supplement; useful as a reference inorganic source in comparative studies	Limited storage; less evidence for superior functional outcomes than organic forms	[24,32,41,42]
Selenium-enriched yeast	Organic Se source, predominantly SeMet in yeast proteins	Absorbed via amino acid pathways; part can enter general body protein pools as a reserve and part supports selenoprotein synthesis	High	High bioavailability; effective increase in blood, milk, and colostral Se; useful in maternal transfer and stress-sensitive stages	More expensive; composition may vary among products	[24,32,36,41,42,43,44]
Selenomethionine (SeMet)	Organic selenoamino acid	Enters amino acid pools; nonspecifically incorporated into body proteins; can later contribute to the functional Se pool	High	Creates a mobilizable reserve; usually more effective than inorganic forms for tissue retention and transfer into milk	Does not itself guarantee direct catalytic incorporation unless converted to the reduced Se pool	[24,32,41,42,44,45]
Hydroxy-selenomethionine/HMSeBA	Organic hydroxy analog/SeMet precursor	Efficiently converted into the SeMet-related pool; supports systemic Se availability	High	Improves plasma and milk Se concentration and antioxidant status more effectively than sodium selenite in dairy cows	Still fewer livestock studies than for Se yeast; species-specific evidence not equally developed	[42,44,45,46]
Selenitetriglycerides	Lipophilic organoselenium compound	Intended to be absorbed with lipids; reported to rapidly increase circulating Se and GPx activity	Probably moderate to high, but still insufficiently characterized	Rapid rise in blood Se; promising maternal–offspring transfer; useful during physiologically demanding periods	Metabolic fate still not fully resolved; comparative livestock evidence remains limited	[27,37,38]
Selenium nanoparticles (nano-Se, SeNPs)	Elemental selenium at nanoscale	Require conversion from elemental Se to reduced Se before entry into selenoprotein biosynthesis	Unclear/formulation-dependent	Lower reported acute toxicity than some conventional forms; promising antioxidant effects	Strong dependence on particle size, coating, aggregation, and synthesis method; poor inter-study comparability	[32,33,35,47,48]
Plant-derived Se sources/Se-enriched plants	Natural organic-dominant matrix; species-dependent Se forms	Delivered within plant matrix; may provide mixed organoselenium forms depending on plant metabolism	Variable	Potential feed-based natural source; relevant in biofortification approaches	Highly dependent on plant species, geography, and Se chemistry in the plant	[25,32,44]

**Table 2:** Representative mammalian selenoproteins relevant to redox control and cellular homeostasis.

Selenoprotein Family/Name	Common Abbreviation (Protein; *Gene*)	Primary Subcellular Localization	Biological Functions and Physiological Roles
Glutathione peroxidases			
Glutathione peroxidase 1	GPx1; *GPX1*	Cytosol, mitochondria	Highly abundant; reduces H_2_O_2_ to water; limits oxidative damage and apoptosis [22,60,62,71,72]
Glutathione peroxidase 2	GPx2; *GPX2*	Cytosol, nucleus	Gastrointestinal-enriched; protects mucosal epithelium from oxidative injury and inflammation; supports intestinal regeneration [21,29,54,62,73]
Glutathione peroxidase 3	GPx3; *GPX3*	Extracellular (plasma)	Secreted enzyme; reduces extracellular hydroperoxides; supports nitric oxide bioavailability; protects renal and cardiovascular tissues [21,54,59,62,74]
Glutathione peroxidase 4	GPx4; *GPX4*	Mitochondria, nucleus, membranes	Reduces phospholipid hydroperoxides in membranes; central inhibitor of ferroptosis; supports sperm structural integrity and motility [21,54,55,59,75]
Glutathione peroxidase 6	GPx6; *GPX6*	Olfactory epithelium	Detoxifies H_2_O_2_; high expression in the olfactory system and embryonic tissues (selenoprotein in humans/pigs; cysteine homolog in rodents) [22,60,68,76,77]
Thioredoxin reductases			
Thioredoxin reductase 1	TrxR1; *TXNRD1*	Cytosol, nucleus	Regenerates reduced thioredoxin; regulates redox-sensitive transcription factors (NRF2, NF-κB); supports DNA synthesis and proliferation [21,22,76,78]
Thioredoxin reductase 2	TrxR2; *TXNRD2*	Mitochondria	Maintains mitochondrial redox homeostasis; limits mitochondria-mediated apoptosis; contributes to detoxification of reactive aldehydes [21,22,76,79]
Thioredoxin–glutathione reductase	TGR (TrxR3); *TXNRD3*	Testis	Expressed in spermatids; reduces thioredoxin and glutathione pools; supports disulfide remodeling during sperm maturation [22,54,80]
Iodothyronine deiodinases			
Iodothyronine deiodinase 1	DIO1 (D1); *DIO1*	Membranes (liver, kidney, thyroid)	Activates thyroid hormone by converting T4 to T3; contributes to systemic T3 homeostasis [22,54]
Iodothyronine deiodinase 2	DIO2 (D2); *DIO2*	ER membrane (brain, muscle, pituitary)	Local T4-to-T3 conversion; supports neurodevelopment, thermogenesis, and muscle regeneration [22,54,81,82]
Iodothyronine deiodinase 3	DIO3 (D3); *DIO3*	Membranes (brain, placenta, skin)	Inactivates T4 and T3 (e.g., to reverse T3); protects developing tissues from excess thyroid hormone signaling [22,54,59]
Selenoprotein P	SeP; *SELENOP*	Plasma (secreted by liver)	Major selenium transport protein; contains multiple Sec residues (up to 10); provides extracellular antioxidant protection, including against lipid hydroperoxides [21,22,25,83]
ER-resident selenoproteins			
Selenoprotein F (15 kDa)	SELENOF (Sep15); *SELENOF*	ER lumen	Protein folding quality control; interacts with glycoprotein folding pathways (e.g., UGGT-associated processes) [22,55,68,69]
Selenoprotein K	SELENOK; *SELENOK*	ER membrane	Supports Ca^2+^ flux during immune cell activation; contributes to ER-associated degradation (ERAD) of misfolded proteins [22,68,71,81,82]
Selenoprotein M	SELENOM; *SELENOM*	ER lumen	Neuroprotective oxidoreductase; modulates Ca^2+^ signaling; limits oxidative and ER stress-associated apoptosis [22,68]
Selenoprotein N	SELENON (SepN1); *SELENON*	ER/sarcoplasmic reticulum	Regulates Ca^2+^ homeostasis via modulation of Ca^2+^-handling proteins (including SERCA-associated pathways); essential for muscle development and maintenance; linked to rigid spine muscular dystrophy when mutated [21,65,68,76,81]
Selenoprotein S	SELENOS (VIMP); *SELENOS*	ER membrane	Component of ERAD; supports retrotranslocation of misfolded proteins; modulates inflammatory signaling and cytokine production [22,68]
Selenoprotein T	SELENOT; *SELENOT*	ER	Regulates Ca^2+^ homeostasis and neuroendocrine secretion; supports ER homeostasis and cellular stress resilience [22,59,64,68]
Other selenoproteins			
Selenoprotein H	SELENOH; *SELENOH*	Nucleus	DNA-binding oxidoreductase; supports antioxidant gene programs (including glutathione-related pathways); contributes to stress resistance [6,22,81]
Selenoprotein I	SELENOI; *SELENOI*	ER/Golgi	Ethanolamine phosphotransferase involved in phosphatidylethanolamine biosynthesis; supports membrane formation and neural development [22,68,82]
Selenoprotein O	SELENOO; *SELENOO*	Mitochondria	Mitochondrial redox-associated protein with kinase-like features; implicated in AMP transfer (AMPylation) of protein substrates [6,22,81]
Selenoprotein V	SELENOV; *SELENOV*	Testis (cytosol)	Testis-enriched; proposed roles in redox regulation during spermatogenesis and lipid metabolism [22,59,68,76]
Selenoprotein W	SELENOW; *SELENOW*	Cytosol (muscle, brain)	Highly expressed in muscle; supports myoblast redox balance and Ca^2+^ channel regulation; implicated in differentiation-related processes [21,71,81,84]
Selenophosphate synthetase 2	SPS2 (SEPHS2); *SEPHS2*	Cytosol, nucleus	Generates selenophosphate from selenide and ATP; required for Sec biosynthesis and selenoprotein production [22,31,76]
Methionine sulfoxide reductase B1	MSRB1 (SelR); *MSRB1*	Cytosol, nucleus	Reduces methionine-R-sulfoxide in proteins; contributes to repair of oxidative protein damage and regulation of immune cell function [22,62,68]

Abbreviations: ER, endoplasmic reticulum; GPx, glutathione peroxidase; SeP, selenoprotein P; Sec, selenocysteine; TGR, thioredoxin–glutathione reductase; TrxR, thioredoxin reductase. Species note: Table 1 summarizes conserved mammalian selenoproteins relevant to cattle, pigs, sheep, and goats.

**Table 3:** Major reported physiological and production effects of different selenium forms in cattle.

Selenium Form	Main Domains Investigated	Major Reported Physiological Effects	Production-Related Outcomes	References
Inorganic selenium (sodium selenite/selenate)	Transition period physiology, immunity, milk Se transfer	Supports selenium status and antioxidant enzyme activity; improves neutrophil function and immune response; participates in regulation of oxidative stress during the periparturient period	Maintains baseline selenium status; limited tissue storage and milk selenium enrichment compared with organic sources	[41,63,85,103,110]
Organic selenium (selenium-enriched yeast/SeMet)	Lactation, immunity, reproductive performance, passive transfer	Improves antioxidant capacity and immune responses; increases selenium incorporation into milk and colostrum; enhances selenium transfer to calves	Increased milk selenium concentration; improved passive immunity in calves; reduced somatic cell count in some studies	[100,101,103,107,108,109]
Hydroxy-selenometionine (HMSeBA)	Oxidative stress, lactation, selenium metabolism	Increases systemic selenium availability and antioxidant status; enhances selenoprotein activity	Higher plasma and milk selenium concentrations compared with sodium selenite; improved antioxidant status under oxidative stress conditions	[45,105,106]
Selenitetriglycerides	Periparturient supplementation, redox response	Rapid increase in blood selenium concentration; increased erythrocyte GPx activity; modulation of hepatic inflammatory and metabolic gene expression	Potential improvement in systemic selenium status during transition period	[15,27,37]
Combined Se + vitamin E supplementation	Transition period physiology, oxidative stress	Synergistic antioxidant action; improved redox balance and immune function during oxidative stress	Improved postpartum reproductive performance and mitigation of transition stress	[29,91,111]

**Table 4:** Major reported physiological and production effects of different selenium forms in pigs.

Selenium Form	Target Physiological Stage	Major Reported Physiological Effects	Production or Health Outcomes	References
Inorganic selenium (sodium selenite/selenate)	Gestation, lactation, nursery and growing pigs	Maintains selenium status and selenoprotein synthesis; supports antioxidant defense	Baseline selenium supply; generally less efficient maternal–offspring transfer compared with organic forms	[11,24,125,127,129]
Organic selenium (selenium-enriched yeast/SeMet)	Sows, neonatal piglets, weaned piglets	Improves antioxidant capacity, immune responses, and selenium retention in tissues; enhances maternal transfer of selenium to offspring	Improved piglet antioxidant status and immune parameters; better resistance to oxidative stress	[36,113,117,126,129]
Selenitetriglycerides	Pregnant sows and offspring	Increased maternal and neonatal selenium concentrations and GPx activity	Improved selenium transfer to piglets during the periparturient period	[38]
Plant-derived organic selenium sources	Weaned piglets	Improves intestinal morphology, antioxidant status, and immune function under intestinal stress	Enhanced intestinal barrier function and reduced inflammatory responses	[122]
Selenium-enriched functional carriers (e.g., MOS)	Weaned piglets	Supports intestinal barrier integrity and antioxidant capacity	Reduced diarrhea incidence and improved intestinal morphology	[121]
Nano-selenium (experimental)	Reproduction and oxidative stress models	Improved antioxidant status and reproductive parameters in experimental systems	Potential improvement of reproductive performance and oxidative stress tolerance	[32,35,84]

## Data Availability

The original contributions presented in this study are included in the article. Further inquiries can be directed to the corresponding author(s).
